# Effects and mechanisms of iron overload on the proliferation and differentiation of preosteoblastic cells *via* a 3D microsphere culture system

**DOI:** 10.3389/fbioe.2026.1700858

**Published:** 2026-01-22

**Authors:** Yu Pan, Xiaojing Luo, Renfeng Zhao, Chengdong Zhang, Xuwei Luo, Gang Feng, Dongqin Xiao

**Affiliations:** 1 Research Institute of Tissue Engineering and Stem Cells, Department of Clinical Lab, Nanchong Central Hospital, The Second Clinical College of North Sichuan Medical College, Nanchong, Sichuan, China; 2 School of Medicine, University of Electronic Science and Technology of China, Chengdu, China

**Keywords:** 3D culture, cytoskeleton, hydrogel microspheres, iron overload, osteoporosis

## Abstract

**Background:**

Iron overload-related osteoporosis has garnered significant attention, yet its pathological mechanisms remain unclear. Traditional two-dimensional (2D) culture systems often fail to recapitulate the extracellular matrix (ECM) microenvironment, leading to discrepancies between in vitro and in vivo findings.

**Methods:**

We developed a three-dimensional (3D) culture system using methacrylated gelatin (GelMA) microspheres to culture preosteoblastic cells, simulate the bone microenvironment under iron overload conditions, and systematically examine changes in cellular morphology, viability, function, and gene expression.

**Results:**

Iron overload impaired cell viability, induced oxidative stress, and inhibited osteogenesis in both 2D and 3D cultures. However, cells in 3D exhibited enhanced resilience, including reduced ROS levels, higher viability, preserved cytoskeletal integrity, and less apoptosis and G1-phase arrest. Compared to 2D, 3D-cultured cells showed downregulated expression of ITGA1 and ITGB1, decreased adhesion function, and promoted proliferation. Transcriptomics further revealed activation of NF-κB signaling and DNA replication pathways in 3D, while key pathways such as Hippo, focal adhesion, and Wnt were suppressed.

**Discussion:**

The GelMA microsphere-based 3D system provides a physiologically relevant model for studying iron overload. These findings offer not only mechanistic insights but also suggest potential microenvironment-targeted therapeutic strategies for iron overload-associated osteoporosis.

## 1 Introduction

Osteoporosis is a common skeletal disorder characterized by reduced bone mass, deterioration of bone tissue microstructure, and increased bone fragility, leading to a higher risk of fractures and functional impairment (Wu et al., 2023). Affecting more than 200 million individuals globally, osteoporosis has become a major health concern for the elderly population and postmenopausal women (Kumari et al., 2024). Osteoporotic fractures are serious complications of this condition. It is estimated that approximately 50% of women and 20% of men over the age of 50 will experience a fracture during their lifetime (Mornar et al., 2024). Osteoporosis can develop without a clear underlying cause (primary osteoporosis) or secondary to other diseases. While the risk factors are not fully elucidated, several contributors have been identified, including advanced age, female gender, hysterectomy, prolonged inactivity, malnutrition, and long-term use of corticosteroids (Song et al., 2022).

Iron is an essential micronutrient involved in various physiological processes, such as hemoglobin synthesis and oxygen transport (Su et al., 2023). However, excessive iron accumulation in the body can lead to iron overload, a pathological condition with systemic implications. This surplus iron catalyzes the generation of reactive oxygen species (ROS) *via* the Fenton reaction, contributing to the development of diverse pathological conditions (Sun et al., 2021). The resulting oxidative stress can adversely affect multiple tissues and organs, including the liver, heart, and bones (Von Brackel and Oheim, 2024). Iron overload is prevalent in various hematological disorders, including beta-thalassemia, hereditary hemochromatosis, and sickle cell disease (Asmarian et al., 2022; Baschant et al., 2022). Its detrimental effects extend beyond the cardiovascular system to include organs such as the liver and endocrine glands (Pistoia et al., 2024; Radushkevitz-Frishman et al., 2023). A growing body of evidence indicates a strong association between iron overload and compromised bone health. In animal models, iron overload has been demonstrated to result in bone loss and microstructural deterioration (Guo et al., 2024). This process involves both increased bone resorption and impaired bone formation (Passin et al., 2025). Notably, studies have reported that iron overload specifically inhibits the osteogenic differentiation of bone marrow mesenchymal stem cells *in vitro*, without affecting their adipogenic or chondrogenic potential (Yan et al., 2025). Despite these findings, the precise molecular mechanisms through which iron overload contributes to osteoporosis remain incompletely understood.

Previous investigations into the effects of iron overload on preosteoblastic cells have predominantly relied on traditional two-dimensional (2D) culture systems, where cells are seeded on surface-modified plates. However, such 2D models are unable to accurately replicate the spatial architecture of preosteoblastic cells and their native extracellular matrix (ECM) microenvironment, potentially leading to physiologically irrelevant behavioral interpretations (Han et al., 2024; Tevlek et al., 2023). Mechanistic studies have identified ferroptosis as a critical regulatory process in iron overload-induced osteoporosis (Liu et al., 2022). Key mediators of ferroptosis, such as upregulation of ACSL4 and downregulation of GPX4, have been shown to inhibit osteogenic differentiation and exacerbate bone loss (Jiang et al., 2024). In murine models of estrogen withdrawal-induced iron overload, iron accumulation was associated with elevated levels of 4-hydroxynonenal (4-HNE), a toxic aldehyde generated by lipid peroxidation, further supporting the role of ferroptosis in bone mass reduction (Ruan et al., 2024). Nevertheless, these findings remain constrained by the limitations of 2D culture, which fails to recapitulate key ECM biophysical properties (e.g., stiffness, porosity) and *biochemical* signaling cues (e.g., integrin-mediated adhesion) (Khanmohammadi et al., 2025; Stadelmann et al., 2022). As a result, pathological mechanisms have been largely attributed to cell-autonomous responses, overlooking the complexity of the native bone microenvironment. The inability of 2D systems to support three-dimensional (3D) growth also leads to diminished cell–cell and cell–matrix interactions (Doray et al., 2021), thereby restricting the development of native morphological and functional phenotypes. Therefore, 3D culture models are necessary to more faithfully simulate physiological conditions and enable accurate assessment of iron overload effects. Emerging autologous cell-based 3D systems have been shown to overcome the limitations of both 2D monolayers and preclinical animal models by better emulating the *in vivo* microenvironment (García-Posadas and Diebold, 2020; Troka et al., 2022). For bone tissue research, such 3D models are expected to provide pivotal insights into bone remodeling mechanisms, thereby advancing pathophysiological and therapeutic studies of skeletal disorders.

Recent advances in 3D cell culture systems have significantly enhanced cell proliferation while preserving native cellular phenotypes and biological functions (Gao et al., 2022; Pais et al., 2021). The ECM, as the structural foundation for cell growth, constitutes an essential component of the cellular microenvironment. It not only provides physical support and mediates signal transduction but also offers anchoring points that sustain cell migration and prevent inhibit apoptosis (Alves et al., 2024; Guberman et al., 2020). *In vivo*, the ECM supplies diverse physical cues, including matrix stiffness, pore size, and microarchitecture (Sampayo et al., 2023; Sethi et al., 2021). Given the complexity of the native ECM, a wide range of materials has been explored as ECM-mimetic substrates. Among these, hydrogels stand out as promising biomaterials due to their inherent capacity to facilitate the diffusion of oxygen, nutrients, and water-soluble metabolites. Their physical and chemical properties can be tailored to closely resemble those of natural tissues (Walker et al., 2021; Zheng et al., 2024). To further improve hydrogel scaffold performance, various strategies have been developed to engineer novel biointerfaces that replicate key aspects of the ECM (Unagolla and Jayasuriya, 2022). The large surface area provided by 3D scaffolds enhances cell-matrix interactions. Natural ECM proteins such as collagen (Li et al., 2025) or gelatin (Kim et al., 2024) are commonly used in microsphere fabrication, as they contain intrinsic bioactive motifs and cell-adhesive sequences that support cellular attachment, proliferation, and osteogenic differentiation. Therefore, gelatin-based microspheres represent promising cell carriers and offer a powerful platform for investigating 3D cell behavior and mechanobiological mechanisms (Li et al., 2021; Miao et al., 2023).

To investigate the mechanism underlying the effect of iron overload on osteoporosis, this study intends to employ gelatin-based microspheres as a 3D cell culture scaffold, and systematically examine the impacts of iron overload on the growth of preosteoblastic cells, thereby providing a theoretical basis and experimental support for the significance of the ECM microenvironment for cell behavior as well as its implications for bone health research related to iron metabolism.

## 2 Materials and methods

### 2.1 Preparation of gelatin with light-curing property

Firstly, 20 g of gelatin (Type A, 300 bloom, porcine skin; Sigma-Aldrich) was dissolved in 200 mL of phosphate-buffered saline (PBS) at 40 °C under continuous stirring for 12 h. Then, 16.0 mL of methacrylic anhydride (Sigma-Aldrich) was added dropwise at 45 °C under constant stirring while protected from light. After reacting for 3 h, the reaction was quenched by adding a five-fold volume of PBS. The mixture was then dialyzed against deionized water using dialysis membranes (MWCO 10–14 kDa) for 7 days. Finally, the purified solution was filtered through a 0.22 μm membrane and lyophilized to obtain GelMA. Finally, the purified solution was filtered through a 0.22 μm membrane and lyophilized to obtain GelMA. The degree of methacryloyl substitution (DS) was calculated according to the literature (Li et al., 2016) by comparing the ^1^H nuclear magnetic resonance Spectroscopy (NMR, Qone-WNMR-I-AS400) NMR signal integrals of the lysine methylene protons in GelMA and unmodified gelatin, using equation:

$$\text{DS }\%=1-\frac{\left(lysine\;methylene\;proton\;of\;GelMA\right)}{\left(lysine\;methylene\;proton\;of\;Gelatin\right)}\times 100\%$$

### 2.2 Preparation of GelMA microspheres

For the preparation, 1 g of GelMA was first dissolved in 10 mL of ultrapure water under constant stirring (500 rpm) at 25 °C until complete dissolution. Then, 0.3 wt% LAP (EFL, China) was added to the aqueous phase, while paraffin oil (EFL, China) served as the oil phase. Monodisperse droplets were generated using a microfluidic device (EFL, China) and photopolymerized *via* 405 nm blue light irradiation at the microfluidic chip outlet. The microspheres were alternately washed with PBS and 75% (v/v) ethanol for three times to remove residual paraffin oil, yielding GelMA microspheres. Then, the microspheres were lyophilized for 24 h and stored at −20 °C. For use, the microspheres were immersed in culture medium in a well plate and incubated at 37 °C for 30 min to achieve swelling equilibrium. Subsequently, the microspheres were soaked in 75% ethanol for 45 min and then washed by PBS for three times to obtain sterile GelMA microspheres.

### 2.3 Physicochemical characterizations

Of the microspheres was measured with an electronic universal testing machine (Model E43.504, MTS Systems Corporation, China; 20N-50 kN load cell), and the modulus was automatically calculated from load-indentation curves using Optics11 Data Viewer V2.0 software (Lee et al., 2016). Data from three independent batches were summarized in a scatter plot generated with GraphPad Prism 9.5 software.

### 2.4 Cell culture

Mouse preosteoblastic cells (MC3T3-E1 subclone 4; ATCC) were cultured in α-minimum essential medium (α-MEM; Gibco, USA) supplemented with 10% fetal bovine serum (FBS) and 1% penicillin/streptomycin (P/S). Cells were maintained in a humidified incubator at 37 °C with 5% CO_2_ and passaged upon reaching 80%–90% confluence.

For experiments involving microspheres, lyophilized GelMA microspheres were sterilized in 75% (v/v) ethanol for 30 min, washed extensively with PBS, and then transferred to agarose-coated low-attachment plates. They were immersed in complete α-MEM overnight prior to cell seeding. Preosteoblastic cells were seeded onto the microspheres (GM group) or directly onto standard tissue culture plates (TCP group) at a density of 10,000 cells per well in a 24-well plate. To simulate iron overload, ferric ammonium citrate (FAC; Sigma-Aldrich) was added to the culture medium at the indicated concentrations, creating the GM + FAC and TCP + FAC treatment groups, respectively. The culture medium was refreshed every other day.

### 2.5 Cell proliferation and viability

Cell proliferation was assessed using the Cell Counting Kit-8 (CCK-8; Kaiji Biotech, China) assay after 1, 3, 5, 7, 9, and 11 days of culture. At each time point, the culture medium was replaced with a mixture of serum-free α-MEM and CCK-8 reagent (10:1 v/v). Following incubation at 37 °C for 2 h, the absorbance of 100 μL of the resulting solution was measured at 450 nm using a microplate reader.

To evaluate the cytotoxic effect of FAC, cells were treated with 0, 50, or 300 μM FAC. Cell viability was measured by the CCK-8 assay after 24, 48, and 72 h of treatment. Additionally, after 48 h of FAC exposure, live/dead staining was performed using 2 μM calcein-AM (for live cells; Invitrogen, USA) and 4 μM propidium iodide (PI; for dead cells; UElandy, China). Cells were imaged using a confocal microscope (Nikon, Japan). Cell viability was quantified by calculating the ratio of calcein-AM-positive cells to the total number of cells (calcein-AM and PI-positive) using ImageJ software.

### 2.6 Apoptosis detection

To assess the effect of FAC treatment on apoptosis, cells were harvested after 2 days of culture and analyzed using an apoptosis assay kit (Elabscience, China). Cells were resuspended in 2.5 μL Annexin V-FITC and 2.5 μL PI (50 μg/mL; Elabscience), gently vortexed, and incubated in the dark at room temperature for 15 min. Next, 400 μL of 1× Annexin V binding buffer was added. Fluorescence intensity was quantified *via* flow cytometry, and apoptosis rates were analyzed with FlowJo v10.8.1.

### 2.7 Cell cycle

Cell cycle analysis was performed using a cell cycle detection kit (Kaiji Biotech, China). After drug treatment, cells were seeded at 1 × 10^5^ cells/well. Cells were harvested, adjusted to 1 × 10^6^ cells/mL in single-cell suspension, and 1 mL of the suspension was processed. Following centrifugation, the supernatant was discarded, and cells were fixed with ice-cold 70% ethanol. Fixed cells were stored overnight at 4 °C, washed with PBS, and stained per the manufacturer’s protocol. Red fluorescence (excitation: 488 nm) was measured *via* flow cytometry, and cell cycle phases were analyzed using ModFit LT v4.1.

### 2.8 Determination of ROS generation

After 2 days of treatment with 300 μM FAC, intracellular ROS levels per cell were quantified through dual-fluorescence normalization. First, nuclei were stained by incubating with 5 μg/mL Hoechst 33342 (Beyotime, China) at 37 °C in the dark for 30 min, followed by three washes with PBS. Subsequently, a ROS-sensitive probe (10 μM DCFH-DA, Solarbio, China) was loaded *via* 30 min dark incubation. After final PBS rinses, TCP cells were detached with 0.25% trypsin-EDTA, whereas GM cells were harvested using ice-cold GelMA lysis buffer. Fluorescence (Hoechst: Ex/Em 350/461 nm; DCF: Ex/Em 488/525 nm) was measured with SpectraMax® iD3 Multi-Mode Microplate Reader (Molecular Devices, USA). ROS levels were expressed as the DCF/Hoechst fluorescence ratio (AU).

### 2.9 Adhesive function of cells

After 2 days of 300 μM FAC treatment, samples were fixed, permeabilized, and blocked. F-actin and nuclei were stained with iFluor™488-phalloidin (1:200) and Hoechst 33342, respectively. Images were acquired using a confocal microscope (Nikon), and cell spreading area was quantified with ImageJ. Immunostaining for ITGA1 and ITGB1 was performed on FAC-treated 2D and 3D samples. After incubation with primary antibodies (1:200; Abcam) and Alexa Fluor® 555-conjugated secondary antibody (1:200; Abcam), samples were co-stained with phalloidin and Hoechst 33342. Images were captured by confocal microscopy. After 2 days of FAC treatment, mRNA expression levels of genes related to cytoskeletal organization and mechanosensing, including ITGA1, ITGB1, YAP1, DBN1, RAP1GAP2, and CDH2, were analyzed *via* RT-qPCR. Corresponding primer sequences are listed in Supplementary Table S1.

### 2.10 Osteogenic differentiation of cells

To evaluate osteogenic potential under different microenvironments, cells were pretreated with 300 μM FAC for 2 days. TCP group cells were induced directly, while GM group cells were digested and reseeded before induction. Osteogenic induction medium was refreshed every 2 days. After 7 days, alkaline phosphatase (ALP) activity was quantified using a microplate assay kit (Beyotime), and collagen deposition was assessed by Sirius Red staining. Calcium nodule formation was evaluated by Alizarin Red S staining after 14 days, with absorbance measured after dissolution. After 7 days, osteocalcin (OCN) and osteopontin (OPN) expression was detected by immunofluorescence staining using specific antibodies and counterstained with phalloidin and Hoechst 33342. Gene expression of OPN and OCN was analyzed by RT-qPCR after 14 days of induction, normalized to Gapdh.

### 2.11 RNA-seq analysis of cells

Total RNA was extracted from cells, and transcriptome sequencing was performed at OE Biotech (Shanghai, China). The Illumina Novaseq 6000 platform was used to obtain the gene expression profile, followed by quality control. The data were mapped to a reference genome using the HISAT2 program. Gene expression levels were quantified using the HTSeq-count program and reported as FPKM (fragments per kilobase of transcript per million mapped reads). Correlation analysis was performed to assess the consistency of gene transcription across different groups, with Pearson’s correlation coefficient |*r*| > 0.9 indicating high stability and sensitivity of gene expression. Differentially expressed genes (DEGs) were identified using DESeq2, with the thresholds set as |Log2FC| > 1 and *p <* 0.05 for significance. Hierarchical clustering was conducted using R (v3.2.0) to visualize gene expression patterns across different groups and samples. Volcano plots were generated to show the distribution of upregulated and downregulated DEGs. Enrichment analysis of key pathways and biological processes was performed using Gene Ontology (GO) and Kyoto Encyclopedia of Genes and Genomes (KEGG) databases to reveal the biological significance of DEGs. Representative DEGs from downregulated signaling pathways were displayed in a heatmap.

### 2.12 Statistical analysis

All cell experiments were performed in triplicate. Data are presented as the mean ± standard deviation (SD). Comparisons between groups were analyzed using one-way or two-way analysis of variance (ANOVA, GraphPad Prism 9.5), and Tukey’s test was applied for *post hoc* comparisons. A *p*-value < 0.05 was considered statistically significant.

## 3 Results and discussion

### 3.1 Characterization of the GelMA microspheres

**FIGURE 1:**
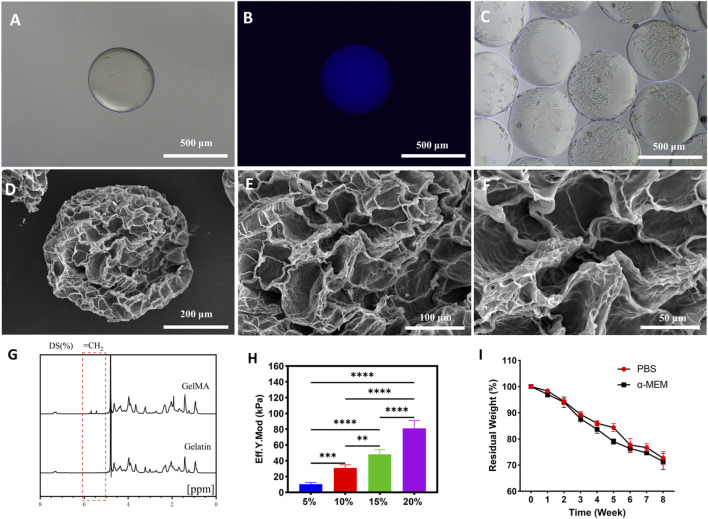
Characterization of the hydrogel microspheres. **(A)** Bright-field image of microspheres in culture medium. **(B)** Fluorescence images of microspheres in culture medium. **(C)** Bright-field image of cells cultured on the microspheres. SEM images of freeze-dried microspheres at magnifications of **(D)** ×500, **(E)** ×1,000 and **(F)** ×2,000. **(G)** NMR spectra of Gelatin and GelMA. **(H)** Young’s modulus of the microspheres (*n* = 10). **(I)** Degradation of microspheres in the culture medium and PBS (*n* = 10).

GelMA microspheres were synthesized using a microfluidic device, with low-magnification stereomicroscopy confirming their uniform spherical morphology and smooth surfaces (Supplementary Figure S1). Direct observation in culture medium further showed the formation of spherical microspheres with a uniform diameter of 500 ± 50 μm (Figure 1A), which exhibited autofluorescence (Figure 1B) and supported cell attachment on their surfaces (Figure 1C). After lyophilization, the surface morphology and internal pore structure of the hydrogel were examined by SEM. The microspheres were spherical (350 ± 50 μm in diameter) with uniform pores (20–50 μm) (Figures 1D–F). Upon cell seeding, light microscopy showed that cells initially formed aggregates in suspension, which then gradually attached to and spread on the microsphere surfaces, eventually forming confluent monolayers. In this study, LAP was chosen as the photoinitiator to crosslink GelMA because of its high water solubility, efficient polymerization under cytocompatible visible light (405 nm), and rapid hydrogel-forming capability while maintaining high cell viability, which make it particularly suitable for 3D cell culture (He et al., 2023).

NMR spectroscopy confirmed the successful synthesis of GelMA. For NMR analysis, the phenylalanine peak (7.1–7.4 ppm) was used as an internal reference in all samples due to its insensitivity to methacrylamide modification. Characteristic peaks at 5.3 and 5.5 ppm, corresponding to the acrylic protons (2H) of methacrylated groups, emerged exclusively in GelMA spectra. Peak intensity showed a positive correlation with the degree of methacrylation, with the integration-based calculation giving a substitution degree of 64.7%, which corresponds to a moderately substituted GelMA product.

Mechanical testing revealed that the Young’s modulus of GelMA increased in a concentration-dependent manner. Specifically, under crosslinking conditions with 30 s irradiation using an ultraviolet lamp (EFL-LS-1600-405, EFL, China), the Young’s moduli of 5% GelMA, 10% GelMA, 15% GelMA, and 20% GelMA were measured as 10.53, 31.03, 48.16, and 81.12 kPa, respectively. Given that the Young’s modulus of tissues with early bone injuries (e.g., osteoporosis) is approximately 35 kPa, while that of tissue culture plates exceeds 3 GPa (Gao et al., 2023; Greaney et al., 2025) with its Young’s modulus far greater than that of *in vivo* tissues, 10% GelMA was selected for microsphere fabrication. Degradation experiments showed that GelMA microspheres exhibited a 30% degradation rate after 8 weeks of immersion in culture medium, with no significant difference in degradation between PBS and α-MEM solutions. This indicated that the microspheres possessed the capacity to support cell loading and mimic the *in vivo* ECM mirenvironment.

### 3.2 Iron overload impairs proliferation and elevates ROS in microsphere-cultured cells

**FIGURE 2:**
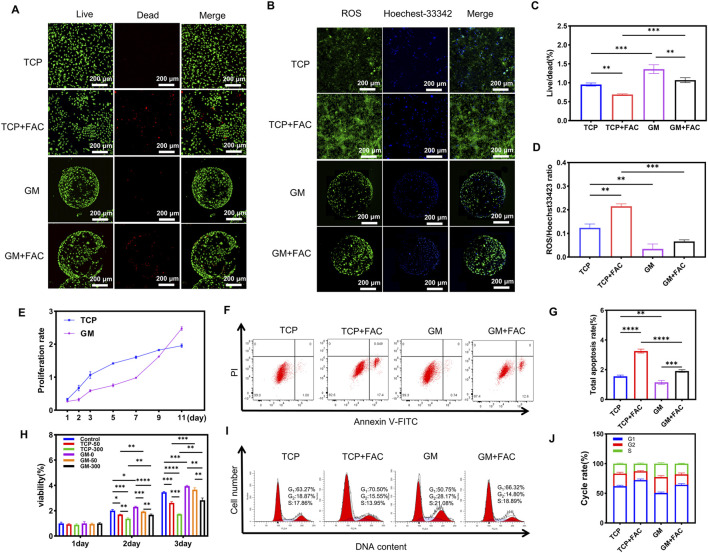
The 3D microsphere culture system attenuates proliferation and ROS expressions in preosteoblastic cells under iron overload. **(A)** Live/dead staining of cells cutured on different groups. **(B)** Intracellular ROS staining of preosteoblastic cells cultured on different groups. **(C)** The corresponding quantitative analysis of live/dead fluorescence. **(D)** The corresponding quantitative analysis of fluorescence intensities. **(E)** Growth curve of preosteoblastic cells cultured on TCP and GM groups with α-MEM without FAC. **(F)** The corresponding quantitative analysis of ratio of early to late apoptosis. **(G)** Apoptosis analysis of preosteoblastic cells cultured on different groups *via* Annexin V-FITC/PI flow cytometry. **(H)** Cell viability of preosteoblastic cells exposure to 50/300 μM FAC for 24/36/72 h. **(I)** Cell cycle distribution of preosteoblastic cells cultured on different groups *via* flow cytometry. **(J)** The corresponding quantitative analysis of cell cycle phase proportions (G1/G2/S). *p < 0.05; **p < 0.01; ***p < 0.001; ****p < 0.0001.

Live/dead staining revealed predominantly green fluorescence with negligible red fluorescence in both TCP and GM groups of the control group, indicating near-complete viability. Following 300 μM FAC treatment for 2 days, red fluorescence intensity significantly increased in TCP + FAC and GM + FAC groups, with TCP + FAC exhibiting substantially stronger red signal than GM + FAC (Figure 2A). After culture for 1 day, cells formed monolayers with complete microsphere coverage and entered active proliferation. At 7 days, the proliferation rate of the GM group was significantly higher than that of the TCP group (Figure 2E). As a methacrylated derivative of gelatin, GelMA retains collagen-derived RGD sequences (arginine-glycine-aspartic acid), which promote cell adhesion through integrin-mediated (Kim et al., 2024). Meanwhile, GelMA microspheres simulate the physicochemical properties of the ECM *via* a 3D porous structure, providing a biomimetic microenvironment for mechanical support, nutrient gradient distribution, and cell-matrix interactions. To investigate the effect of iron overload on cell growth, preosteoblastic cells cultured on TCP and GM were treated with 0 (control group), 50, or 300 μM FAC for 24, 48, and 72 h. This concentration range was selected to effectively mimic *in vitro* iron overload and elicits distinct biological responses in osteoblastic cells (Jiang et al., 2022). On 2 days, cell viability in the TCP group was 330% ± 3.1% in the control group, 256% ± 2.8% at 50 μM, and 207% ± 3.5% at 300 μM, while the GM group showed 390% ± 2.9%, 355% ± 3.3%, and 270% ± 2.5% viability under the same conditions (Figure 2H). The GM group exhibited significantly higher resistance to iron overload-induced cytotoxicity, likely due to the in vivo-like microenvironment simulated by the 3D culture system. The quantitative results were normalized to the TCP control group, with values of 100% ± 1.2%, 70% ± 2.1%, 130% ± 3.8%, and 95% ± 1.9% for the TCP control group, TCP + FAC group, GM group, and GM + FAC group, respectively. Notably, GM + FAC demonstrated significantly higher viability than TCP + FAC (Figure 2C), indicating that the 3D ECM microenvironment enhances cellular resistance to iron toxicity compared to 2D culture. These results are consistent with Hildenbrand et al. (2024), who reported weaker inhibition of tumor cell proliferation by iron-doped bioactive glasses in 3D cultures than in 2D systems, suggesting that 3D microenvironments enhance cellular resistance to iron toxicity by *modulating adhesion signaling*, and *metabolic* pathways. Based on these findings, 300 μM FAC treatment for 2 days was selected to simulate iron-overloaded pathological conditions.

Fluorescence imaging revealed distinct patterns of ROS generation between 2D and 3D culture systems under iron overload (Figure 2B). In TCP group, 300 μM FAC treatment induced intense and diffusely distributed ROS fluorescence compared to the control group. By contrast, GM group exhibited minimal baseline ROS signals, and FAC treatment triggered only spatially restricted ROS accumulation that was substantially weaker than that in 2D culture. Nuclear staining with Hoechst-33342 confirmed comparable cellular density across all groups, supporting the use of normalized ROS/Hoechst-33342 fluorescence ratios for quantitative comparisons. TCP cells showed a 75% increase in the ROS/Hoechst-33342 ratio (from 0.12 ± 0.02 AU to 0.21 ± 0.03 AU; *p* < 0.01), whereas GM cells exhibited only a 50% increase (from 0.03 ± 0.01 AU to 0.06 ± 0.02 AU; *p* < 0.05) (Figure 2D). Notably, normalized ROS levels in GM microspheres remained significantly lower than those in TCP cells under both basal and iron-overloaded conditions (*p* < 0.01).

Flow cytometry analysis revealed significantly attenuated apoptosis in GM group cells under iron overload. Total apoptosis rates were quantified as: 1.00% ± 0.12% (TCP), 17.45% ± 1.32% (TCP + FAC), 0.74% ± 0.08% (GM), and 12.60% ± 0.95% (GM + FAC). Notably, GM + FAC demonstrated 28% lower apoptosis than TCP + FAC (*p* < 0.0001), confirming superior iron toxicity resistance in the 3D microenvironment (Figures 2F,G). Flow cytometry showed that iron overload prolonged the G1 phase in both TCP + FAC and GM + FAC groups, consistent with previous (Yanagiya et al., 2025), indicating cell cycle arrest. However, the GM group exhibited a higher proportion of cells in the S phase than the TCP group, suggesting that 3D culture promotes proliferation by extending the S phase. Swethaa and Ravi (2023) demonstrated that GM group cells have prolonged S phase and logarithmic growth phase, delaying proliferation arrest, which may allow more time for DNA replication and repair. Chen and Dong (2025) showed that the physical properties of 3D scaffold influenced cyclin expression *via integrin-cytoskeleton* signaling.

### 3.3 The effects of iron overload on cell-ECM interactions in preosteoblastic cells cultured on microspheres

**FIGURE 3:**
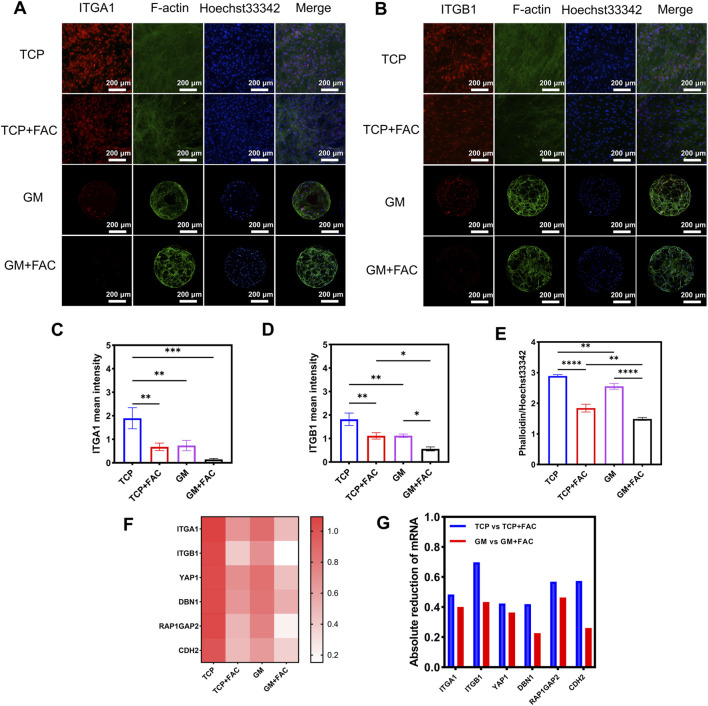
The 3D microsphere culture system attenuates integrin-mediated mechanotransduction in preosteoblastic cells under iron overload. The immunofluorescence staining of **(A)** ITGA1 and **(B)** ITGB1 of cells cultured on different groups. And the corresponding quantitative analysis of the mean fluorescence intensity (MFI) for **(C)** ITGA1, **(D)** ITGB1 and **(E)** F-actin. **(F)** The mechanotransduction-related gene expressions of cells cultured on different groups. **(G)** The corresponding quantitative analysis of the absolute reduction of mRNA expression for mechanotransduction-related genes. *p < 0.05, **p < 0.01, ***p < 0.001, ****p < 0.0001.

Integrins are a class of important cell surface adhesion molecules formed by the heterodimerization of alpha and beta subunits. They collectively form the integrin α1β1 complex, which is primarily responsible for mediating adhesion between cells and the ECM. This complex plays a crucial role in various cellular physiological processes, including cell adhesion, migration, and signal transduction. Immunofluorescence stainings (Figures 3A,B) revealed distinct actin-integrin architectures in different groups. In 2D culture, cells on TCP exhibited dense, planar F-actin networks with intense membrane-localized ITGA1/ITGB1 signals, indicative of robust focal adhesion-dependent adhesion. In contrast, exposure to FAC in the TCP + FAC group resulted in fragmented F-actin and a drastic reduction in ITGA1/ITGB1 fluorescence intensity (Figures 3C,D), reflecting severe cytoskeletal and adhesion disruption. Conversely, cells in the GM groups displayed an interwoven 3D F-actin meshwork with substantially lower ITGA1/ITGB1 expression than those on TCP, consistent with previous reports that 3D culture reduced reliance on ITGA1/ITGB1-mediated adhesion (Zhou et al., 2017). Notably, following FAC treatment, cells on microspheres maintained partial integrity of their 3D actin reticulum, accompanied by a significant downregulation of ITGA1/ITGB1 compared to the TCP + FAC group.

Previous studies have indicated that cell adhesion is primarily associated with the Hippo signaling pathway, integrin-mediated cell adhesion signaling pathway, and the Rap1 regulatory 2022) (Ahmad et al., 2022). Among these, YAP1 plays a critical role as a key factor in cell adhesion within the Hippo signaling pathway, where it regulates the transcription of downstream target genes through nuclear translocation, thereby influencing biological processes such as cell adhesion, proliferation, and differentiation (Ahmad et al., 2022). The actin assembly factor DBN1 serves as a downstream target gene of YAP1, promoting F-actin polymerization and stabilizing the actin cytoskeleton (Krauss, 2017). Together, they form the YAP1-DBN1-actin signaling axis, which provides a structural basis for the stable formation of focal adhesions mediated by integrins ITGA1 and ITGB1 on the cell membrane. The Rap1 regulatory pathway maintains the dynamic balance of adhesion functions by modulating the activation state of ITGA1 and ITGB1. Additionally, the adhesion molecule CDH2 (N-cadherin) participates in this regulatory network by promoting intercellular connections and dynamically coordinating with ITGA1 and ITGB1 to regulate the dynamic changes of focal adhesions (Revollo et al., 2015). The transcription of CDH2 is also indirectly regulated by YAP1, creating a bidirectional feedback loop that integrates mechanical signals with the expression of adhesion molecules. Therefore, this study further explores the expression levels of these cell adhesion-related genes.

RT-qPCR analysis (Figure 3F) demonstrated that YAP1, DBN1, CDH2, RAP1GAP2, ITGA1, and ITGB1 were expressed at significantly higher levels in the TCP group than in the 3D microsphere (GM) group. Following FAC treatment, expression of all six genes was markedly downregulated in both groups. However, the absolute decrease in expression was more gradual in the GM + FAC group compared to the sharp decline observed in the TCP + FAC group. This indicated that the 3D culture system mitigated the rapid iron overload-induced decline in adhesion-related gene expression. The expression of RAP1GAP2, a negative regulator of Rap1 GTPase, correlated with the cellular response to iron overload. Its transcript level was markedly reduced in the TCP + FAC group but was relatively maintained in the GM + FAC group, showing an inverse relationship with the severity of iron overload damage. We hypothesize that the sustained expression of RAP1GAP2 within the 3D microenvironment may contribute to the observed cytoprotection. The maintained RAP1GAP2 expression may preserve integrin function and cytoskeletal integrity by restraining Rap1-dependent integrin hyperactivation, a process likely dysregulated in the 2D system under stress. Consequently, the sustained expression of RAP1GAP2 may represent a mechanism by which the 3D system buffers iron overload stress, a hypothesis that awaits functional confirmation.

The results above indicate that iron overload can interfere with the expression of adhesion-related genes, subsequently affecting the expression and function of ITGA1/ITGB1, ultimately leading to cytoskeletal disorganization and impaired adhesion function. However, when iron overload occurs within a 3D culture system, this destructive chain reaction is significantly alleviated. This suggests that the 3D structure may regulate the fluctuations in the expression of adhesion-related genes and maintain the structural stability of the cellular microenvironment, thereby supporting the functional homeostasis of ITGA1/ITGB1 and protecting the cytoskeleton from iron overload-induced damage.

### 3.4 The effects of iron overload on osteogenic capability in preosteoblastic cells cultured on microspheres

**FIGURE 4:**
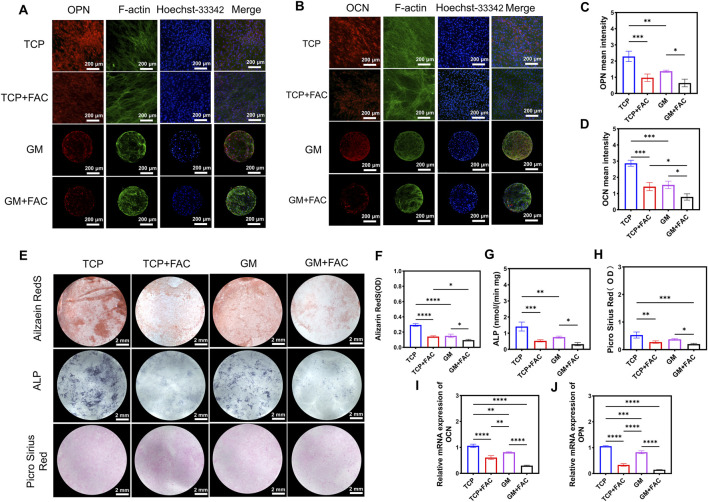
The 3D microsphere culture system attenuates osteogenic function impairment in preosteoblastic cells under iron overload. The immunofluorescence staining of **(A)** OPN and **(B)** OCN of cells cultured on different groups. And the corresponding quantitative analysis of the MFI for **(C)** OPN, **(D)** OCN. **(E)** Alizarin Red (calcium nodule formation), alkaline phosphatase (ALP, early osteogenic marker), and Sirius Red (collagen deposition) staining. Quantification of **(F)** Alizarin Red staining, **(G)** ALP staining, **(H)** Sirius Red. **(J–I)** The osteogenic - related gene expressions of cells cultured on different groups. *p < 0.05, **p < 0.01, ***p < 0.001, ****p < 0.0001.

Iron overload was observed to exert a more pronounced inhibitory effect on osteogenic differentiation in 2D TCP cultures than in 3D GelMA microspheres. Markedly reduced signals of OPN and OCN were detected by immunofluorescence staining in TCP cultures following FAC treatment (Figures 4A,D). Although decreased OPN and OCN fluorescence was also observed in GelMA cultures after FAC exposure, the signal intensity was maintained at a level significantly higher than that measured in TCP cultures, indicating that the iron-induced suppression of these osteogenic markers was attenuated by the 3D matrix.

Substantially diminished mineralization was demonstrated by Alizarin Red S staining in TCP cultures under iron overload (Figures 4E,F). Similarly, strong reductions in early osteogenesis and collagen deposition were revealed by measurements of ALP activity and Picrosirius Red staining, respectively, in TCP + FAC cultures (Figures 4E,G,H). In contrast, significantly greater mineralized area, ALP activity, and collagen content were maintained in GelMA cultures despite iron challenge, although these values were found to be lower than those in untreated GelMA controls.

At the transcriptional level, pronounced downregulation of OPN and OCN mRNA expression was shown by RT‒qPCR analysis in TCP cultures exposed to FAC (Figures 4I,J). However, significantly higher levels of both transcripts were preserved in GelMA cultures under identical conditions. These findings indicated that osteogenic differentiation was suppressed by iron overload through reduction of OPN and OCN expression accompanied by impaired mineralization and collagen synthesis. These detrimental effects were substantially mitigated by the 3D GelMA microenvironment, which was consistent with its previously documented capacity to maintain cell viability, reduce oxidative stress. These results demonstrated that GelMA microspheres established a protective microenvironment, which allowed for bone regeneration under iron-overload conditions.

### 3.5 RNA-seq analysis of preosteoblastic cells cultured on microspheres under iron-overloaded condition

Whole transcriptome RNA sequencing was carried out to further investigate the effects of 2D and 3D culture systems under iron overload condition. For cells cultured in TCP and GM groups, volcano plots (as shown in Supplementary Figure S2A) and hierarchical clustering heatmap (Supplementary Figure S2B) showed the significantly differentiated gene distribution between the TCP and GM groups. Compared to TCP group, 3,312 genes were significantly upregulated and 3,545 significantly downregulated in GM. GO and GSEA enrichment analyses (Supplementary Figures S2C–L) indicated that compared to TCP group, the biological processes of the upregulated genes in GM group were enriched in *cell cycle regulation*, *DNA replication*, and *chromosome segregation*, while the downregulated genes were mainly enriched in *focal adhesion*, *actin cytoskeleton regulation*, *ECM-receptor interaction*, and *the Hippo signaling pathway*. All these results indicated that compared to TCP, the GelMA microspheres could alleviate cytoskeletal tension and inhibit adhesion-mediated signal transduction.

**FIGURE 5:**
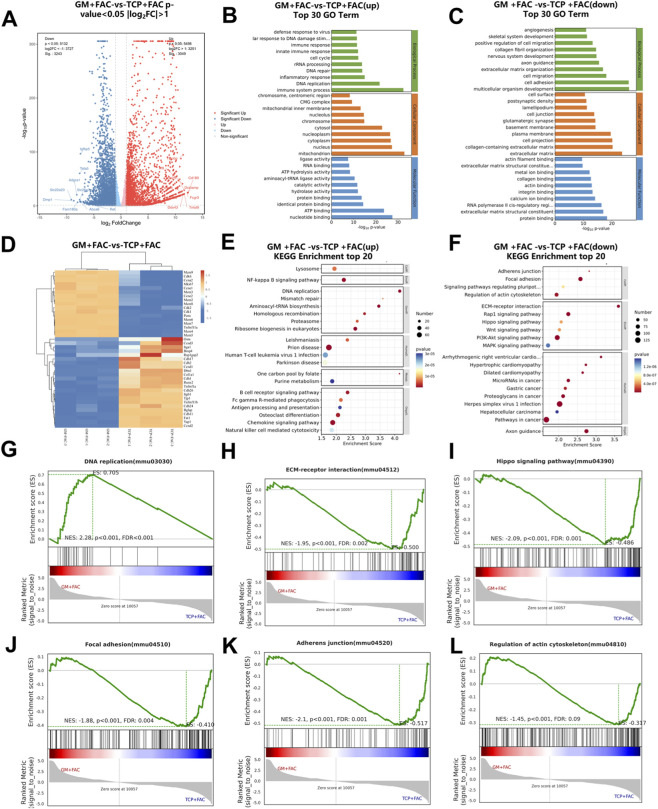
RNA sequencing analysis of preosteoblastic cells cultured on GM + FAC and TCP + FAC. **(A)** Volcano plot of DEGs between GM and TCP groups. **(B,C)** KEGG pathway enrichment analysis of upregulated and downregulated genes. **(D)** Cluster heatmap of DEGs in GM and TCP, with low expression indicated in blue and high expression in orange. **(E,F)** GO enrichment analysis of upregulated and downregulated genes between the two groups. **(G–L)** GSEA enrichment analysis of inter-group signaling pathways.

Under iron overload condition (GM + FAC vs. TCP + FAC), Compared to TCP + FAC, 3,049 genes were significantly upregulated and 3,243 significantly downregulated in GM + FAC (Figure 5A). Similarly, cell cycle-related genes (Cdk, Ccnd, CCNA) and proliferation-associated genes (MCM, PCNA) remained significantly upregulated in GM + FAC, while osteogenic differentiation genes (Tnfrsf11b, Tnfrsf1a, Bmp4, Bglap, Runx2), cell adhesion-related genes (Cdh, Itga1, Itgb1, Rap1gap2), and Hippo signaling effector molecules (Yap1) were markedly downregulated (Figure 5B). Crucially, the upregulations of *DNA repair* and *NF-κB signaling pathways* in GM + FAC group were further pronounced under iron stress, suggesting that GM + FAC may promote cell growth by leveraging these pre-activated mechanisms to counteract iron toxicity. Downregulated genes remained significantly enriched in biological processes including *skeletal system development*, *regulation of cell migration*, *collagen fibril organization*, and *cell adhesion* (Figures 5C,D). KEGG pathway (Figures 5E,F) analysis further indicated that upregulated genes were significantly enriched in the *NF-κB signaling pathway*, *DNA replication*, and *osteoclast differentiation pathways*, while downregulated genes were mainly associated with *adherens junctions*, *focal adhesions*, *actin cytoskeleton regulation*, *Rap1 signaling*, *Hippo signaling*, *Wnt signaling*, *PI3K-Akt signaling*, and *MAPK signaling pathways.* Notably, quantitative GSEA analysis showed that the transcriptomic advantage established by the 3D microenvironment was robustly maintained rather than compromised by stress. As shown in Figures 5G–L, the normalized enrichment score (NES) values of core pathways closely mirrored the baseline patterns: the *DNA replication pathway* remained highly activated (NES = 2.28, Figure 5G), while *mechanotransduction-related pathways* such as *Focal adhesion* (NES = −1.88, Figure 5J) remained suppressed. Even under the severe perturbation induced by iron overload, the NES values of these pathways remained nearly identical to the baseline levels. All these results indicated that the 3D microenvironment could stably maintain the cell’s high-repair state, thereby preventing iron overload from impairing this intrinsic advantage.

**FIGURE 6:**
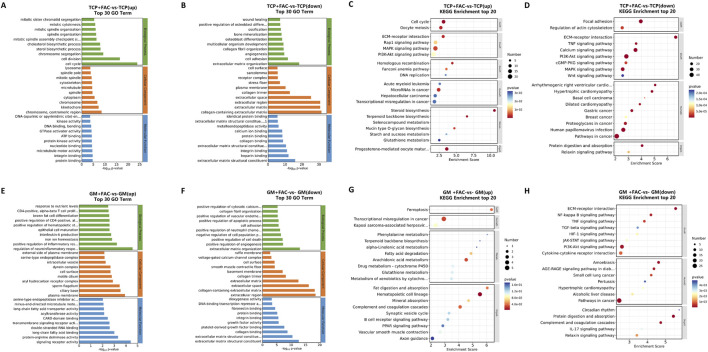
RNA sequencing analysis of preosteoblastic cells cultured in the same culture system (TCP vs TCP + FAC, GM vs GM + FAC). **(A,B)** GO enrichment analysis for upregulated and downregulated genes of TCP + FAC and TCP. **(C,D)** KEGG pathway enrichment analysis for upregulated and downregulated genes of TCP + FAC and TCP. **(E,F)** GO enrichment analysis for upregulated and downregulated genes of GM + FAC and GM. **(G,H)** KEGG pathway enrichment analysis for upregulated and downregulated genes of GM + FAC and GM.

Moreover, the biological function between the same culture systems with and without iron overload (TCP + FAC vs TCP, GM + FAC vs GM) were compared (Figures 6A–D). In 2D cell culture, cell cycle-related pathways (*mitotic chromatid segregation*, *spindle assembly*) and metabolic reprogramming pathways (*cholesterol/sterol biosynthesis*, *glutathione metabolism*) were found to be significantly upregulated in the TCP + FAC group, correlating with enhanced proliferative (Tao et al., 2024). Downregulated signaling pathways were predominantly associated with *osteogenic differentiation (bone mineralization, ossification) and ECM-receptor interaction networks*. Glutathione (GSH) is a redox-active molecule endogenously present in the human body that activates redox enzymes and the enzymatic antioxidant system. It participates in the regulation and homeostasis of intracellular redox reactions, thereby alleviating oxidative stress and maintaining cells in a stable physiological state. The results of this study demonstrated that preosteoblastic cells may counteract the toxicity of iron overload through the reactive upregulation of GSH (Mak et al., 2017; Tian et al., 2020). In the 3D cell culture, compared with the GM group, the upregulated genes were enriched in immune regulatory pathways (*IL-6 signaling, T cell proliferation*) and ferroptosis-related gene sets in the GM + FAC group. Ferroptosis is a form of regulated cell death characterized by iron ion-dependent catalysis: when the intracellular reducing system is inactivated, phospholipids containing long-chain unsaturated fatty acids on the cell membrane or organelle membranes undergo peroxidative damage, ultimately leading to cell membrane rupture. Previous studies have demonstrated that ferroptosis is highly associated with lipid peroxidation and labile iron pool accumulation (Pan et al., 2025; Xia et al., 2019). In the present study, GSEA analysis confirmed that the ferroptosis pathway was significantly enriched in preosteoblastic cells in the 2D culture (Supplementary Figure S3), directly demonstrating that ferroptosis is the core mechanism underlying iron overload induced cell damage in the 2D culture. KEGG enrichment analysis further revealed that iron overload downregulated the *extracellular matrix-receptor interaction* and *focal adhesion pathways* in 2D cultured cells (Figure 6D), while the upregulation of the *glutathione metabolism pathway* (Figure 6C) was insufficient to counteract the activation of ferroptosis. Although ferroptosis-related signals were also detected in 3D cultured cells (Figure 6G), these cells exhibited a more significant upregulation of the *glutathione metabolism pathway*, accompanied by the enrichment of GO terms related to nutrient response and ion homeostasis (Figure 6E). This suggests that 3D culture can enhance the antioxidant capacity of cells through metabolic regulation. Notably, iron overload disrupted the homeostasis of matrix components in both culture systems. KEGG analysis showed that the HIF-1, TGF-β, and *focal adhesion signaling* pathways were inhibited (Figures 6D,H), and genes related to collagen biosynthesis were downregulated (Figures 6B,F). The results of ROS staining, osteogenic differentiation assays, and transcriptomic data indicate that the 3D culture system can regulate integrin-mediated cell adhesion and cytoskeletal remodeling, thereby enhancing the glutathione-dependent antioxidant defense mechanism. This regulatory effect can improve cell proliferation capacity while alleviating iron overload-induced ferroptosis, ultimately leading to GM group cells exhibiting significantly greater resistance to iron toxicity compared with TCP group cells.

**FIGURE 7:**
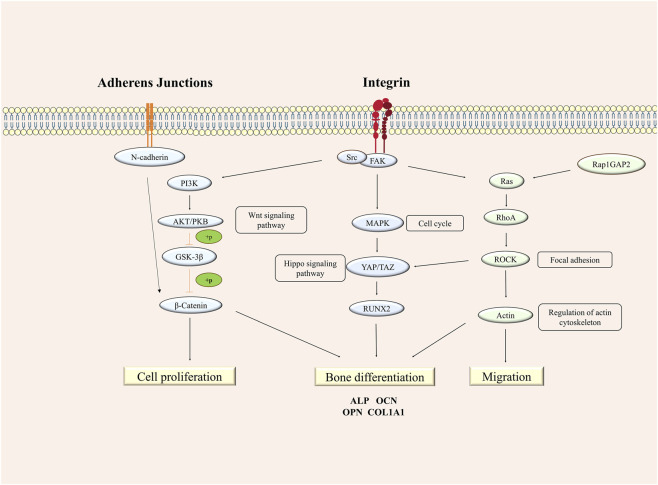
The schematic diagram of integrin mediated-signal pathway regulations on preosteoblastic cells under iron overload in 3D cell culture.

Collectively, markedly different growth behaviors and gene expression profiles were observed in cells within 3D culture environments compared to 2D cultures. These core differences were rooted in the distinct mechanical properties of the culture system and the inherent distinctions in cell-matrix interactions. Firstly, GelMA hydrogels that mimic components of the ECM provided a 3D microenvironment closely resembling *in vivo* conditions. The Young’s modulus of these synthesized microspheres was significantly lower than the Young’s modulus of tissue culture plastic used in 2D cultures (Chen et al., 2021), aligning more closely with the physiological Young’s modulus range of *in vivo* bone tissue (Lee et al., 2016). Concurrently, their porous structure offers favorable conditions for cell adhesion, migration, and nutrient exchange (Ci et al., 2024). Consequently, cells cultured on these microspheres demonstrated advantages such as increased proliferation capacity, reduced ROS accumulation, and maintained greater stability in osteogenic function, experiencing only a more attenuated decline under iron overload, this stands in sharp contrast to the substantial reduction in osteogenic function observed in TCP group cells. In the 2D culture system, the excessively high Young’s modulus of TCP leads to overstretching of the cell cytoskeleton (Chatterjee et al., 2022), while ROS accumulation induced by iron overload exacerbated this damage chain, not only does it cause actin fragmentation and disrupt mechanosignal transduction, but it also disturbs the functional balance of the integrin-focal adhesion kinase (FAK)-mitogen-activated protein kinase (MAPK) axis (Duan et al., 2017). This regulatory imbalance manifests as functional dysregulation characterized by an initial abnormal high expression of osteogenic markers OPN, OCN followed by a rapid downregulation and an increased rate of apoptosis, ultimately leading to anomalies in the cell cycle—specifically, a significant G1 phase arrest in preosteoblastic cells, a marked reduction in the S and G2/M phase ratios, and a resultant sharp decline in proliferation capacity. Conversely, the physiological Young’s modulus of GelMA microspheres within the 3D culture system serves as a core support for cells to resist iron overload-induced damage. This is achieved by remodeling the dynamics of the cytoskeleton (Ren et al., 2023) and enhancing the glutathione-dependent antioxidant system (Sönmez Aydın et al., 2021), thereby maintaining high cell viability while ensuring proliferation functionality (Chen et al., 2022). Transcriptomic analysis reveals that the cell cycle distribution more closely resembles the normal state, characterized by upregulated cyclin D1 and suppressed p21 expression. This aligns with the findings of Chen and Dong (2025) which demonstrate that the physical properties of 3D scaffolds can modulate cell cycle protein expression *via* integrin-cytoskeleton signaling, thereby fine-tuning proliferation and differentiation. Portantly, transcriptomic data from the GM group unequivocally showed downregulated expression of cell adhesion-related molecules (e.g., ITGA1/ITGB1), thereby attenuating cell-ECM adhesion. This moderate adhesion characteristic exerts a synergistic regulatory effect with the physiological hardness of the microspheres: on one hand, it mitigates integrin signaling, preventing excessive nuclear translocation of YAP, which could lead to imbalance osteogenic differentiation (Zhang et al., 2024); on the other hand, it utilizes Rap1GAP2 to inhibit the Rap1 signaling pathway, thereby avoiding excessive activation of integrins while supporting pathways involved in proliferation and antioxidant defense. Simultaneously, the interconnected actin cytoskeleton provides structural support for antioxidant systems (e.g., Nrf2) and promotes moderate nuclear translocation of YAP through nuclear pore dilation or upregulation of BAG3 chaperone protein expression during stress (Günay et al., 2021), establishing a dynamic balance of inhibition-moderate activation to maintain proliferation. The 3D culture system modulates cytoskeletal dynamics, proliferation, and differentiation through the coordinated regulation of multiple signaling pathways. In this network, the adherens junction pathway plays a central role. Three-dimensional culture downregulates N-cadherin expression, thereby weakening intercellular adhesion. This pathway also promotes β-catenin stability and nuclear translocation *via* the PI3K/AKT axis, directly supporting cell proliferation (Zhang et al., 2025). Within the integrin pathway, altered expression of ITGA1 and ITGB1 disrupts downstream signaling and reduces functional synergy with the Hippo effector YAP1, leading to suppressed activity of the transcription factor RUNX2 (Zhang et al., 2024). These molecular changes align with the observed decrease in cell-matrix adhesion and attenuation of osteogenic differentiation. Acting as a mechanical signaling hub, the cytoskeletal migration pathway involves the dynamic regulation of RAP1GAP2 and DBN1, which cooperate with RhoA/ROCK signaling to remodel the actin cytoskeleton (He et al., 2019). By adjusting cytoskeletal tension, this pathway influences both YAP activity and β-catenin signaling in adherens junctions. Through close and dynamic cross-talk, these pathways collectively establish a transcriptional and functional equilibrium in the 3D microenvironment that favors proliferation while permitting adaptive differentiation. This integrated regulatory network explains the enhanced oxidative stress resistance and sustained functional homeostasis observed under iron overload (Figure 7).

Although specific mechanism underlying how the iron overload affecting cell behavior in 3D culture system remains to be further investigated, this study has confirmed the significant differences between 2D and 3D cultures, particularly emphasizing the critical role of cell adhesion in regulating cell function and gene expression. Limitations of this research include that the mechanistic studies have not been fully carried out. Given the sequencing results, future studies could focus on validating the identified differentially expressed genes, confirming their protein-level expression, and further characterizing the associated cell–matrix signaling pathways. Furthermore, future studies should determine whether iron overload mediates cellular behavior through ferroptosis in a 3D cell culture, and elucidate the associated regulatory role of autophagy. Additionally, the incorporation of various osteogenesis-related cells, including osteoblasts, osteoclasts, endothelial cells, and immune cells within this model, could construct osteoporosis-related tissue models, facilitating the assessment of their feasibility for drug screening through *in vivo* and *in vitro* experiments.

## 4 Conclusion

In summary, this study utilized GelMA microspheres to construct a 3D culture platform to investigate the effects of iron overload on the growth and differentiation of preosteoblastic cells. Compared to 2D cultures, the 3D culture system exhibited enhanced resistance to iron toxicity, demonstrating higher survival rates, smaller increases in ROS levels, and moderately downregulated osteogenic differentiation function. These phenomena may be attributed to the regulation of mechanical transduction in the 3D culture system, where specific integrin-mediated signaling pathways between cells and the microenvironment (particularly the ITGA1/ITGB1 pathway) modulate the activity of the YAP/TAZ pathway and activate antioxidant systems, ultimately enabling an adaptive response of cells to iron overload. Although the underlying mechanisms require further elucidation, this study provides a novel research tool based on cell-ECM interactions to explore the role of iron overload in osteoporosis, which may offer deeper mechanistic insights and develop new therapeutic strategies for osteoporosis.

## Data Availability

The original contributions presented in the study are included in the article/Supplementary Material, further inquiries can be directed to the corresponding authors.
