# Supplementary material for: Effects and mechanisms of iron overload on the proliferation and differentiation of preosteoblastic cells via a 3D microsphere culture system
Source: Front Bioeng Biotechnol. 2026 Jan 22;14:1700858. doi: 10.3389/fbioe.2026.1700858 (PMC12872815; doi:10.3389/fbioe.2026.1700858)
Supplement: Supplementary file 1 [file Supplementaryfile1.docx]

| Gene | | Primer Sequence（5’‒3’） | | Length（bp） |
| --- | --- | --- | --- | --- |
| GAPDH | F: GCACAGTCAAGGCCGAGAAT | | 20 | |
|  | R: GCCTTCTCCATGGTGGTGA | | 19 | |
| OCN | F: CAGACCTAGCAGACACCATGAG | | 22 | |
|  | R: CGTCCATACTTTCGAGGCAG | | 20 | |
| OPN | F: CAGAAGCTTTTCCAAGTAAGTCCA | | 24 | |
|  | R: CAGTGACCAGTTCATCAGATTCAT | | 21 | |
| ITGA1 | F: GGAAGACCTGGCTGAAGATG | | 19 | |
|  | R: TCCAGGGTAGGTGTCCTTGT | | 20 | |
| ITGB1 | F: GCCTACTTCTGCACGATGGT | | 20 | |
|  | R: CACACCGTTGTCACCGTAGT | | 20 | |
| YAP1 | F: AGCTCCGAGATGGATGGGTA | | 20 | |
|  | R: TCATGCTTAGTCCACTGGCT | | 20 | |
| DBN1 | F: TGGAGAGCCTCAACAACAAG | | 20 | |
|  | R: TCCAGCATCTCCTTCACCTC | | 20 | |
| RAP1GAP2 | F: GAGGACCTGGTGAAGAAGGC | | 20 | |
|  | R: GTCGTAGCCGTAGTCCTTGG | | 20 | |
| CDH2 | F: TGCCAACTGGAATCGATACCG | | 21 | |
|  | R: GGAGTTATGGGGGCATTGAC | | 20 | |

Table S1. Primer sequences used for RT-qPCR


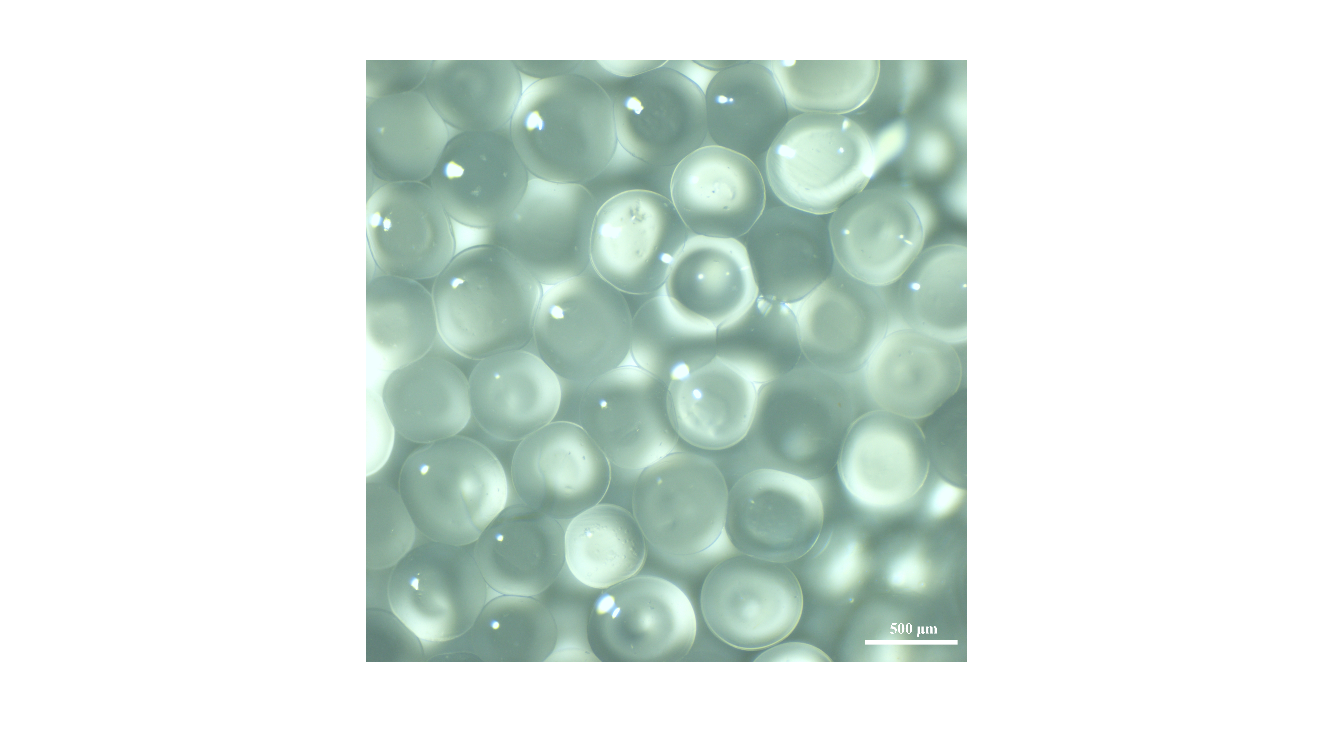


Fig. S1. Morphological characterization of GelMA microspheres. Representative bright-field microscopy image showing the uniform spherical structure and smooth surface of GelMA microspheres under low magnification (4×).


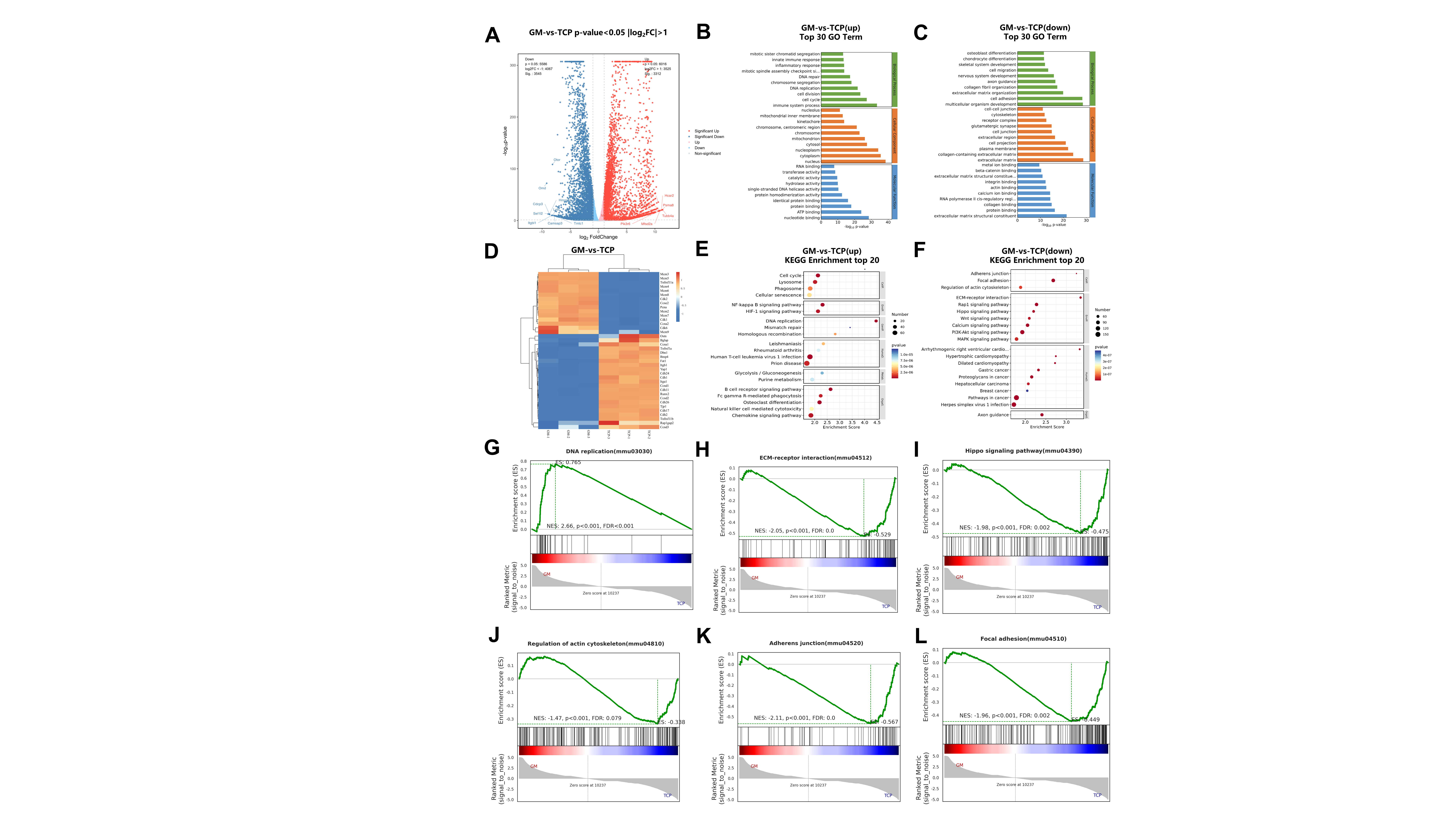


Fig. S2 RNA sequencing analysis of Preosteoblastic cells cultured on GM and TCP. (A) Volcano plot of DEGs between GM and TCP groups. (B-C) KEGG pathway enrichment analysis of upregulated and downregulated genes. (D) Cluster heatmap of DEGs in GM and TCP, with low expression indicated in blue and high expression in orange (n = 3 for each group). (E-F) GO enrichment analysis of upregulated and downregulated genes between the two groups. (G-L) GSEA enrichment analysis of inter-group signaling pathways.


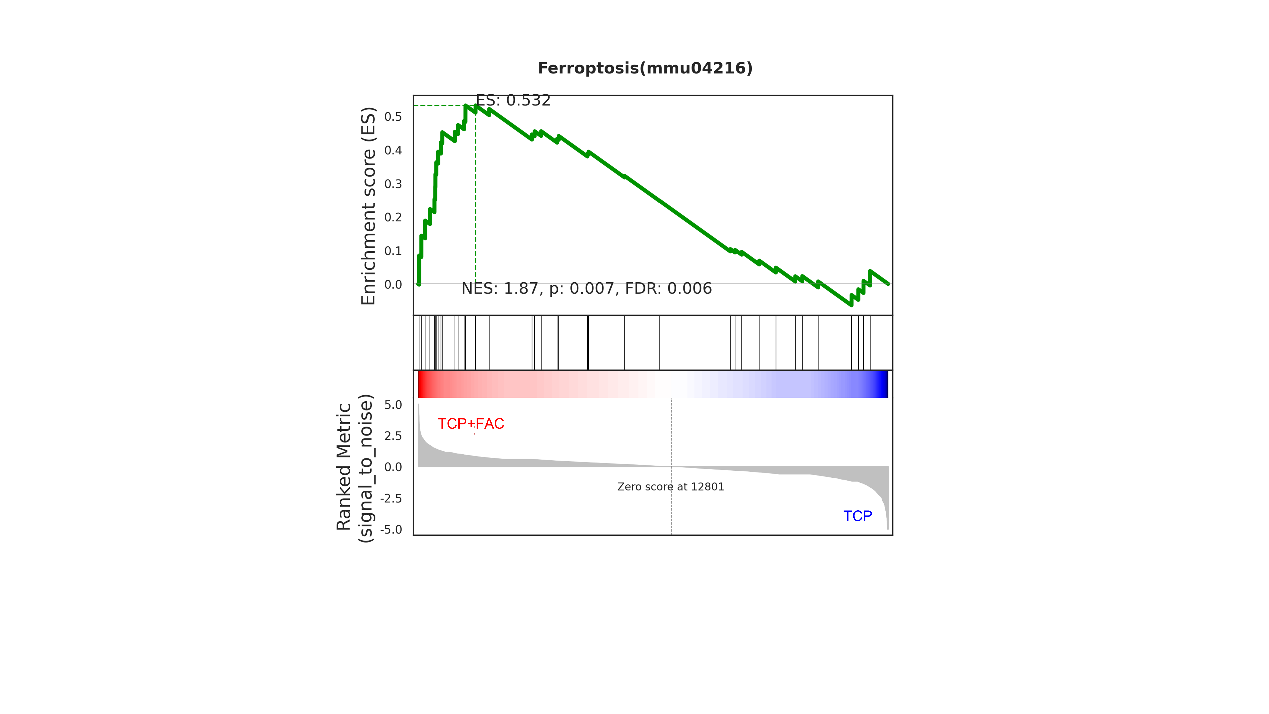


Fig. S3 GSEA enrichment analysis of TCP and TCP+FAC.
